# Management of Patients with Treatment of Pheochromocytoma: A Critical Appraisal

**DOI:** 10.3390/cancers14163845

**Published:** 2022-08-09

**Authors:** Florence Bihain, Claire Nomine-Criqui, Philippe Guerci, Stephane Gasman, Marc Klein, Laurent Brunaud

**Affiliations:** 1 Department of Gastrointestinal, Visceral, Metabolic, and Cancer Surgery (CVMC), University Hospital of Nancy (CHRU Nancy), University of Lorraine, Rue du Morvan, 54511 Vandoeuvre-les-Nancy, France; 2 Inserm UMR_S1256 Nutrition-Genetics-Environmental Risk Exposure, University of Lorraine, 54500 Nancy, France; 3 Department of Anesthesiology and Critical Care Medicine, Institut Lorrain du Coeur Et Des Vaisseaux, University Hospital of Nancy (CHRU Nancy), University of Lorraine, Rue du Morvan, 54511 Vandoeuvre-les-Nancy, France; 4 Centre National de la Recherche Scientifique, Institut des Neurosciences Cellulaires et Intégratives, Université de Strasbourg, 67000 Strasbourg, France; 5 Department of Endocrinology, Diabetology, and Nutrition (EDN), University Hospital of Nancy (CHRU Nancy), University of Lorraine, Rue du Morvan, 54511 Vandoeuvre-les-Nancy, France

**Keywords:** pheochromocytoma, management of pheochromocytoma, alpha blockade, preoperative medical preparation, intraoperative hemodynamic instability, mini-invasive surgery, adrenalectomy

## Abstract

**Simple Summary:**

Preoperative medical preparation with an alpha blockade has been implemented early 1960s to prevent intraoperative hemodynamic instability and consequently decrease morbi-mortality in patients at a high risk of complications. Indeed, surgery at that time consisted of open adrenalectomies with a high morbidity and mortality rate. Current clinical guidelines are based on these early experiences. However, emerging technologies have permitted a drastic reduction of the morbi-mortality rate in patients treated for pheochromocytoma. However, the clinical guidelines have not evolved at the same rate. The aim of this systematic review is to assess the evolution of the management of pheochromocytomas and to appraise the current clinical guidelines to the current clinical practices.

**Abstract:**

The management of pheochromocytomas has significantly evolved these last 50 years, especially with the emergence of new technologies such as laparoscopic procedures in the 1990s. A preoperative blockade using antihypertensive medications to prevent intraoperative hemodynamic instability and cardiocirculatory events is recommended by current clinical guidelines. However, these guidelines are still based on former experiences and are subject to discussion in the scientific community. The aim of this systematic review was to assess the evolution of the management of pheochromocytomas. Laparoscopic procedure is established as the standard of care in current practices. Preoperative medical preparation should be questioned because it does not significantly improve intraoperative events or the risk of postoperative complications in current clinical practice. Current clinical recommendations should be revised and upgraded to current clinical practices.

## 1. Introduction

Pheochromocytomas and paragangliomas are rare tumors of chromaffin cells responsible for autonomous and unregulated secretion of catecholamines such as norepinephrine, epinephrine or even dopamine [1]. Pheochromocytomas derive from adrenal glands, and paragangliomas—from extra adrenal tissues (sympathetic ganglia in the thorax, abdomen and pelvis and from parasympathetic ganglia in the head and neck) [1]. These two types of tumors do not have the same vascularization, and paragangliomas do not have a main vein draining directly into the vena cava (directly on the right and into the renal vein on the left). For the sake of clarity, we will only discuss pheochromocytomas here. The incidence of pheochromocytoma ranges from 0.005 to 0.1% in the general population [2]. The symptoms are due to excessive and episodic secretion of catecholamines and vary as regards to which catecholamine is predominantly produced. The clinical presentation is not specific and represents a challenge for physicians. The broad spectrum of symptoms comprises headaches, palpitations, sweating, pallor, anxiety, tachycardia and hypertension [3]. Hypertension is mostly paroxysmal, but some patients may present sustained hypertension. Metabolic complications with hyperglycemia, lactic acidosis and weight loss can also be symptoms. Stress, medication, pain, manipulation of the abdomen or the tumor can trigger the symptoms [4]. Hypercatecholaminemia and longstanding hypertension can lead to life-threatening conditions such as congestive heart failure, myocardial infarction, shock, cerebrovascular accidents, hemorrhage or even dissecting aneurysms [4]. Diagnosis requires biochemical testing with search of elevated production of catecholamine metabolites in blood and/or urine and localization of the tumor(s) [5]. Computed tomography and magnetic resonance imaging are the usual imaging techniques used for preoperative localization [5]. However, these techniques lack specificity, and functional imaging is then used to improve tumor characterization with MIBG scintigraphy and increasingly with positron emission tomography using several different ligands (18F-DOPA, 18F-FDG, 68Ga-DOTATATE) [6]. About 10% of pheochromocytoma patients will experience acute complications, particularly acute stress cardiomyopathy (Takotsubo), occurring generally before the diagnosis of pheochromocytoma [7]. These patients are then initially managed in an intensive care unit (intubation, extracorporeal membrane oxygenation...) and are outside the scope of this work. Surgical resection is the only curative treatment for patients with pheochromocytoma [8]. Emerging technologies with the use of laparoscopic techniques has permitted the transition from an open approach, the gold standard since the 1930s, to a minimally invasive approach [9]. At the same time, preoperative imaging, intraoperative anesthetic monitoring, intraoperative care and genetic screening strategies have improved considerably during this period. This transition is responsible for a significant and important reduction in perioperative morbidity and mortality. A preoperative blockade in order to control blood pressure and prevent intraoperative hypertensive crisis has been considered the standard approach for preoperative management since the 1960s when it was shown that with the introduction of alpha blockers before an adrenalectomy, morbi-mortality had severely decreased [8]. The rationale of these preoperative recommendations (preoperative antihypertensive medications and intravenous infusion) has not changed since. However, the management of pheochromocytomas in terms of surgery, anesthetics and preoperative imaging tools has dramatically evolved [10]. The goal of this systematic review was to assess the available literature on the perioperative management of pheochromocytomas with the development of new technologies and to determine if a preoperative blockade was still useful and relevant in the current clinical setting.

## 2. Methods

### 2.1. Systematic Literature Search

The PRISMA guidelines were followed for this systematic review [11]. A search of the electronic databases of MEDLINE (PubMed), Web of science and Cochrane Central of Register of Control trials (CENTRAL) was conducted according to the recommendations [12]. The search strategy in PubMed consisted of searching for key terms such as “adrenalectomy”, “pheochromocytoma”, “management of pheochromocytoma”, “alpha blockade”, “preoperative medical preparation”, “intraoperative hemodynamic instability”, “mini-invasive surgery” and their synonyms. Related Medical Subject Headings (MeSH) were also investigated. This procedure was repeated for the CENTRAL and Web of Science database. This search was conducted by two independent investigators from 1 January 2022 to 14 February 2022.

### 2.2. Study Selection

**Figure 1:**
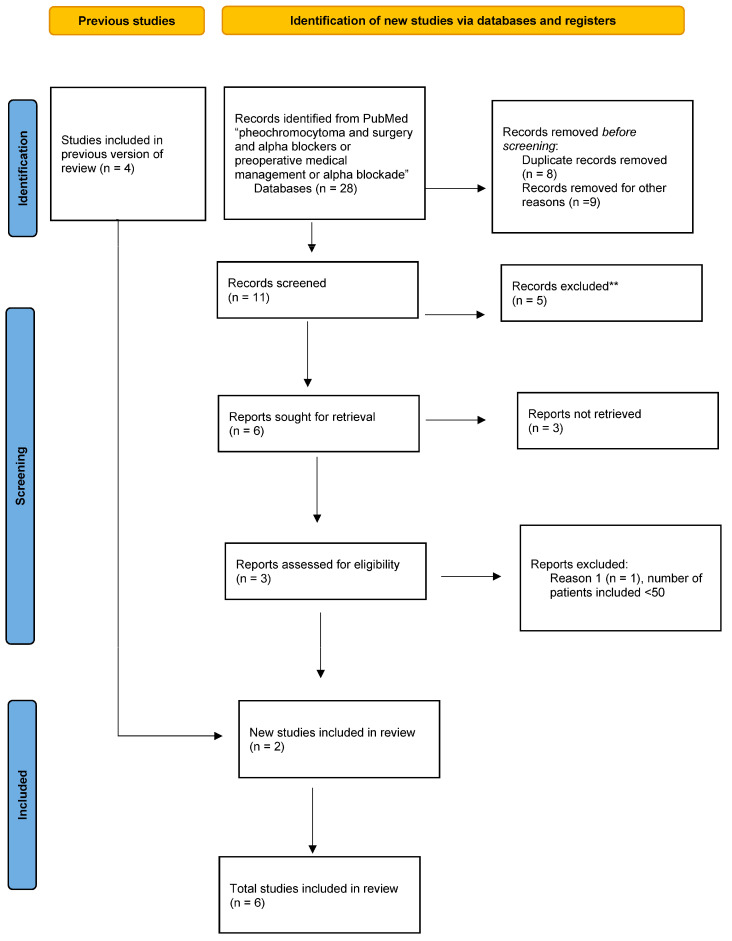
Flow chart according to the PRISMA guidelines: preoperative medical preparation (PMP).

**Figure 2:**
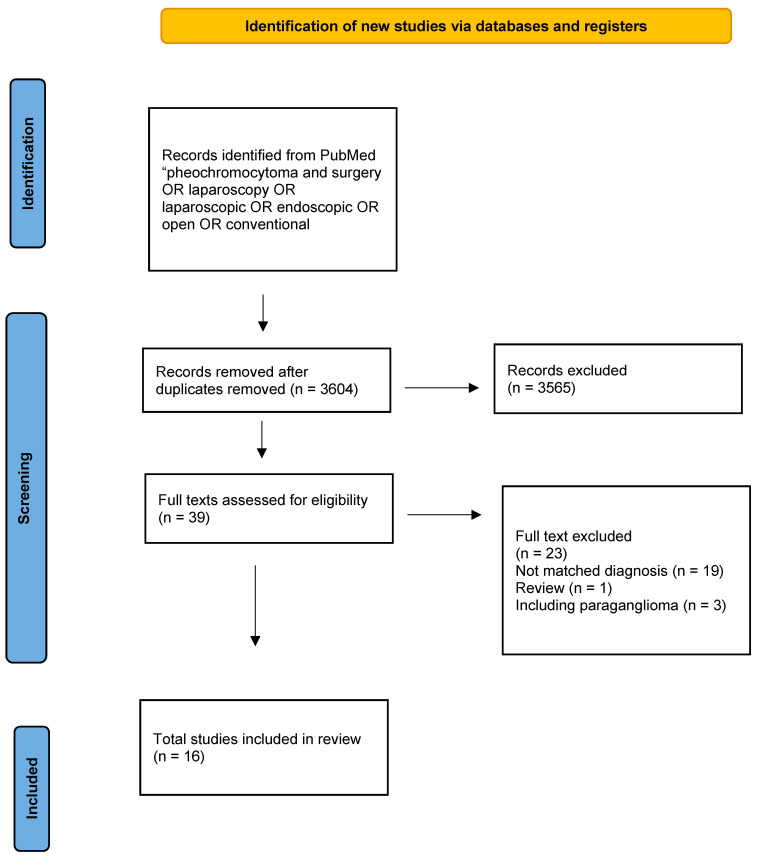
Flow chart according to the PRISMA guidelines: surgical treatment of pheochromocytomas.

Randomized controlled studies (RCT) and non-RCTs evaluating on the one hand surgical treatment of pheochromocytomas and one the other hand preoperative medical preparation (PMP) for patients with pheochromocytoma were eligible for inclusion (Figure 1 and Figure 2). Eligible studies had to have a clinical impact by being published in a journal with a high impact factor ≥3. Meeting abstracts, letters, comments, animal studies, studies in a language other than English and publications for which the whole text could not be salvaged were excluded. Studies on paragangliomas were also excluded from this review because the vascularization of these tumors, hormonal secretion, and intraoperative hemodynamic instability episodes may have biased this study’s conclusions. The quality of the selected studies was revised by the Newcastle-Ottawa scale for the assessment of the quality of nonrandomized studies.

### 2.3. Data Extraction

From each included study, the following criteria were extracted: title, author, year of publication, journal, language, trial design and trial period, number of patients included, treatment groups, loss of follow-up and withdrawals. Outcomes of interest were, on the one hand, operative time, maximum diameter of tumor, estimated blood loss, postoperative hospital stay, complications and conversions between laparoscopic and open procedures. On the other hand, intraoperative hemodynamic instability (maximum systolic blood pressure, intraoperative hypotensions) mortality and morbidity in patients with and without PMP were collected. These outcomes were chosen because of their relevance regarding the aim of this study and their availability in the papers of interest.

## 3. Results

### 3.1. Surgical Treatment for Pheochromocytoma

Adrenalectomy is the standard treatment for pheochromocytomas [13]. Before 1990, the gold-standard treatment of pheochromocytomas was an open adrenalectomy with an anterior transperitoneal or a posterior retroperitoneal incision. Ross et al. describe in 1967 their experiences treating patients with pheochromocytoma [14]. Preoperative diagnostic tools were not as performant in locating the tumor as they are nowadays. Surgery consisted of large open incisions to explore the abdominal cavity. It was not always conclusive, as the tumor was not always found during operation. Extensive resection of organs, such as nephrectomies and splenectomies, were sometimes needed [14]. Perioperative extensive hemorrhage also occurred, causing hemodynamic instability episodes [14]. Surgical management at that time was associated with a high morbi-mortality rate. Riddell et al. in 1963 in their experience with 21 patients with pheochromocytoma rated operative mortality at approximately 30% [15]. Moreover, operative mortality in patients with undiagnosed pheochromocytoma could range up to 50% [15].

The management of pheochromocytoma has significantly changed in current clinical practices. Since the first successful laparoscopic adrenalectomy was performed in 1992, laparoscopic adrenalectomies as a treatment for pheochromocytoma has widely expanded [16,17,18] The 2014 Endocrine Society recommends minimally invasive surgery for most adrenal pheochromocytoma using either transabdominal or retroperitoneal approaches [19]. In 1997, Vargas et al. compared an open approach to the laparoscopic approach in patients with pheochromocytoma [20]. It was shown that there were shorter hospital stays, lower requirement of analgesics and a shorter convalescent period after laparoscopic adrenalectomy. Consequently, laparoscopic approaches were proposed as the new standard of care for the treatment of pheochromocytoma. Schell et al. showed in their study that since the beginning of laparoscopic adrenalectomies in 1992, the open approach was progressively abandoned in favor of the laparoscopic approach, demonstrating the effectiveness and safeness of the laparoscopic procedures [21].

There is no randomized controlled trial comparing the laparoscopic approach to the open approach. However, several studies have evaluated the laparoscopic approach as treatment for pheochromocytoma [22] (Table 1). All of these studies confirm that with a minimally invasive approach, patients had a lower complication rate, shorter operative time, a shorter hospitalization stay and less blood loss [20,21,22,23,24,25,26,27,28,29,30,31,32,33,34,35,36]. More recent studies with a much larger number of patients have confirmed the effectiveness and safeness of a laparoscopic approach compared to those of a laparotomy [26]. Eight recent studies examined a total of 625 patients having underwent an adrenalectomy for pheochromocytoma, in which 328 underwent a laparoscopic adrenalectomy, and confirmed that the complication rate, operative time, hospital stay and blood loss were lesser in the laparoscopic group [21,25,26,27,30,31,32,35]. More specifically, recent reports reported no mortalities after a laparoscopic adrenalectomy for pheochromocytoma in current clinical settings [37,38,39]. Overall, these published reports confirm the trend towards the development and use of minimally invasive technologies with the parallel observation of the virtual disappearance of postoperative mortality and the very significant reduction in complications in patients operated on for pheochromocytoma. Current clinical guidelines state that laparoscopic approaches must be considered as the standard of care for the management of pheochromocytoma.

### 3.2. The Concept of Preoperative Medical Preparation (PMP) to Avoid Intraoperative Hemodynamic Instability

PMP using antihypertensive medications (blockade) was introduced in the fifties when it was shown that without adrenergic blocking agents, adrenalectomy for pheochromocytoma was a dangerous procedure causing a hypertensive crisis, arrythmias, severe fluctuations when manipulating the tumor or even cardiac arrest [14]. From then on, the uncontrolled release of catecholamines in response to anesthetic and surgical stimuli was considered to be responsible for hemodynamic instability (association of hypertensive episodes followed by hypotensive phases) and to favor the occurrence of complications. Goldstein et al. analyzed 104 patients with pheochromocytoma from 1950 to 1998 [40]. In this study, patients received a preoperative blockade from 1967. The importance of routine PMP was considered by the decrease of complications with the use of antihypertensive agents: 69% patients who did not receive an alpha blockade (historical controls) had a complicated surgical course versus 3% in patients treated with phenoxybenzamine (more recent patients). However, in this study, patients were managed using large thoracoabdominal incisions. In four patients, a splenectomy was also associated with the adrenalectomy. Moreover, not all patients had a diagnosis and a precise tumor localization before surgery [40]. In 1967, Ross et al. showed in case reports that the introduction of an alpha-adrenergic blockade controlled preoperative blood pressure, could limit the incidence and severity of intraoperative hypertensive episodes and was associated with preoperative vasodilation allowing re-expansion of contracted plasma volume [14]. It then became a standard of care to treat patients preoperatively with antihypertensive drugs before a resection of pheochromocytoma. In 1983, Roizen et al. showed that in patients who received preoperative treatment using an alpha blockade, systemic and pulmonary pressures did significantly increase with no consequence on oxygen concentration and myocardial function [41]. It was then concluded, even though there were no consequences on oxygen concentration and myocardial function, that an alpha-adrenergic blockade prior to pheochromocytoma excision protected myocardial performance and oxygen delivery [41]. Later, Bruynzeel et al. showed that a risk factor responsible for hemodynamic instability was mean arterial pressure (MAP) at presentation above 100 mmHg [42]. It comforted and implemented the use of an alpha-adrenergic blockade before surgery for pheochromocytoma. Since then, a preoperative blockade has been considered to be associated with reduced episodes of intraoperative hemodynamic instability, resulting in decreased mortality and postoperative complications. It has therefore been incorporated into preoperative management as a standard of care, although the decrease in mortality and morbidity cannot solely be attributed to preoperative management [7].

### 3.3. Medications Used for Preoperative Medical Preparation (PMP)

The rationale of the alpha blockade is to control blood pressure to avoid intraoperative hemodynamic instability and prevent complications due to a massive surge release of catecholamines [3,5]. Phenoxybenzamine is an alpha antagonist that blocks both nonselectively and irreversibly alpha 1 and alpha 2 receptors, therefore obstructing the effects of circulating catecholamines [3]. It has a long duration of action. However, the consequent side effects are associated with the use of phenoxybenzamine: orthostatic hypotension, reflex tachycardia, nasal congestion, syncope and dizziness. Beta antagonist receptors can reduce the reflex tachycardia associated with the use of phenoxybenzamine or other nonselective alpha blockers [3]. Recommendations advocate initiating treatment 7 to 14 days before surgery [3]. Selective alpha blockers, such as doxazosin, can be used in place of phenoxybenzamine [3]. It has the advantage of having a shorter duration of action. It also has the advantage of inducing fewer side effects. Calcium channel blockers have also shown their efficacity as PMP [3]. They are short-acting agents. They can control blood pressure and have benefits such as cardio and renal protection [3]. Calcium channel blockers and alpha blockers have been found to have the same impact on the incidence and severity of intraoperative hemodynamic instability episodes [43,44]. Magnesium has been shown to decrease the release of the catecholamine surge with better control of adrenergic control during surgery for pheochromocytoma [45]. Magnesium has inhibitory action on the catecholamine reuptake by the adrenal gland with reduction in the sensitivity of adrenergic receptors and can directly block catecholamine receptors [45]. No prospective controlled studies have evaluated the use of magnesium in patients with pheochromocytoma. To date, the main drugs used for PMP involve alpha-adrenergic receptor antagonists (nonselective or selective), beta-adrenergic receptor antagonists and calcium channel blockers [4]. Current guidelines recommend a nonselective alpha antagonist (phenoxybenzamine) as the first-line drug to control preoperative blood pressure in patients with pheochromocytoma [3].

### 3.4. Is There a Relationship between Preoperative Blood Pressure Control after Preparation and Perioperative Hemodynamic Events?

There is no consensus on which preoperative cardiac parameters should be obtained after preparation to improve intraoperative hemodynamic instability. In 1987, Roizen et al. established the criteria to reach before surgery to avoid intraoperative hemodynamic instability episodes: BP < 160/90 mmHg for 24 h prior to surgery, no postural hypotension <80/45 mmHg and no ST-T waves changes [41]. Bruynzeel criteria differed from those of Roizen et al. [42]. To avoid intraoperative hemodynamic instability, blood pressure after PMP should be lower than 130/85 mmHg and mean arterial pressure should be <100 mmHg [42]. There is no current consensus on the target hemodynamic parameters to be achieved after PMP, making the preoperative blood pressure goal vague and imprecise, and especially not useful for studies evaluating the potential relation between blood pressure after PMP and IHI episodes (incidence and severity).

In addition, another remaining problem is that the definition of an episode of intraoperative hemodynamic instability is not consensual in the available literature (Table 2). In a recent systematic review investigating risks factors for perioperative hemodynamic instability in pheochromocytoma regrouping 14 studies involving 1725 patients, there were 14 different definitions for intraoperative hemodynamic instability [46]. This demonstrates the lack of uniformity in the scientific community and the strain of comparing studies using different definitions for intraoperative hemodynamic instability.

Beyond these definitional issues, the relationship between target blood pressure after preparation and the risk of occurrence of episodes of hemodynamic instability also remain controversial. Lentschener et al. were among the first teams to question the significant association between preinduction blood pressure and intraoperative hemodynamic fluctuations [47]. A multicenter retrospective study compared three different classes of hypertensive agents used for PMP, selective alpha blockers, nonselective alpha blockers and calcium channel blockers in reducing intraoperative hemodynamic instability [43]. It was shown that preoperative mean systolic blood pressure was lower with the alpha blockade. However, intraoperative hemodynamic instability was independent of which antihypertensive medication was used [43]. In this study, severe hypotensive episodes were more associated with the alpha blockade. As a consequence, the use of intraoperative vasoactive drugs and intraoperative fluid volume were increased in patients who benefited from the alpha blockade [43]. Namekawa et al. investigated clinical factors inducing intraoperative and postoperative hypotension [48]. All of the patients underwent a proper PMP with alpha blockers, and if tachycardia was present, beta blockers were added. This study showed that half of their patients required postoperatively a continuous infusion of catecholamines to maintain blood pressure above 90 mmHg [48].

In a systematic study, tumor size and levels of urinary norepinephrine were associated with perioperative hemodynamic instability [46]. However, the use of alpha-blocking agents for preparation was not associated with intraoperative hemodynamic instability [46]. On the contrary, Kierman et al. used the definition of SBP > 200 mhg, use of vasoactive drugs and heart rate (HR) > 110 bpm for intraoperative hemodynamic instability [49]. Using that definition, they showed that tumor size, the use of a selective blockade and open adrenalectomy were associated with intraoperative hemodynamic instability. A recent meta-analysis compared selective vs nonselective alpha blockers before surgery [50]. They included eleven studies with 1344 patients. Patients treated with selective drugs had a higher intraoperative systolic blood pressure and a lower minimum systolic blood pressure. This meta-analysis concluded that nonselective alpha blockers were more effective in preventing intraoperative blood pressure fluctuations with the same risk between both selective and nonselective drugs in terms of intraoperative and postoperative hypotension [50].

In 2020, a meta-analysis showed that peak systolic and diastolic blood pressure and heart rate during an adrenalectomy for pheochromocytoma were not different in patients with and without alpha-blocker preparation [51]. A recent randomized control trial compared nonselective and selective drug alpha blockades to reduce intraoperative hemodynamic instability [39]. In this study, Buitenwerf et al. showed that there was no difference between selective and nonselective drugs in terms of duration of blood pressure outside the target range during resection. They also studied the intraoperative hemodynamic instability based on a score (IHI score) which included several different components: blood pressure, heart rate, cumulative of dosage of vasoactive medication and fluid therapy. They demonstrated that nonselective alpha blockers were more effective in reducing the IHI score (38.0 (28.8–58.0) in the nonselective group versus 50 (35.3–63.8) in the selective group, *p* = 0.02).

In conclusion, some risk factors associated with intraoperative hemodynamic instability are nonmodifiable, such as tumor size and catecholamine levels. No study has shown that the use of a specific type of hypertensive medication during preparation can prevent the occurrence of hypertensive and hypotensive episodes during surgery. Some studies have suggested that the more effective and nonselective the drug used to control blood pressure, the lower the observed intraoperative hypertensive peaks but the more important the hypotensive phases are likely to be.

### 3.5. Is There a Link between Perioperative Hemodynamic Episodes and Postoperative Complications?

The link between intraoperative hemodynamic instability and complications during and after surgery is central to the principle of medical preparation before pheochromocytoma surgery. However, data showing a significant association between intraoperative blood pressure fluctuations and complications are almost nonexistent in the current clinical setting. A retrospective study including 225 patients and analyzing predictors of morbidity in five international medical centers showed that only two definitions of intraoperative hemodynamic instability correlated with the occurrence of postoperative complications: SBP >= 160 mmHg + MAP < 60 mmHg and SBP >= 200 mmHg + MAP < 60 mmHg [37]. However, a correlation does not mean causation, and this same study reported that blood pressure normalization after PMP and before induction had no impact on postoperative morbidity [37]. In 2020, a meta-analysis conducted by Schimmack et al. evaluated the benefit of PMP before an adrenalectomy for pheochromocytoma [51] (Table 3). In this study, the authors only included studies in which the quality of evidence passed the standards of the Grading of Recommendation Assessment, Development and Evaluation system. Four studies met the inclusion criteria and evaluated the efficacity of a preoperative blockade versus no blockade before adrenalectomy. It was concluded that no difference existed between the blockade versus no blockade in terms of mean SBP, HR, cardiovascular complication or mortality [51]. In the same matter, Groeben et al. conducted a multicenter review of perioperative management and outcomes for pheochromocytoma regrouping data from international centers. In this review that assembled data from 21 centers with a total of 1860 included patients, it was shown that there was no difference between an alpha blockade versus no blockade in terms of intraoperative SBP and mortality [52]. Several other studies do not support a possible link between blood pressure fluctuations and complications during or after adrenalectomy. A meta-analysis including 1344 patients compared selective vs nonselective alpha blockers before surgery [50]. This study concluded that morbidity did not differ between the selective and nonselective group patients [50]. Another study compared 276 patients treated with preoperative alpha blockers versus 156 with other antihypertensive drugs or no treatment [52]. As no significant complications occurred in both groups, the authors concluded that it was necessary to question the role of preoperative preparation in preventing complications.

In the end, most of the available studies against and also historical studies in favor of the preparation present similar methodological biases. All these studies are retrospective, without a control group and with a retrospective analysis of complications observed in operated patients (inducing a risk of under- or overestimation bias) [7]. In one of the only available prospective randomized trials, Buitenwerf et al. confirmed the absence of a significant association between intraoperative hemodynamic instability and intra- and postoperative clinical complications [39]. However, the authors acknowledge that their study was not structured to assess this criterion and was underpowered. Because of the rarity of complications observed during and after an adrenalectomy for pheochromocytoma in current clinical practice, it is likely that no prospective, controlled study will ever be feasible [38]. It was estimated that several thousand patients would have to be included in a randomized study to show a possible difference in complications between two types of medical preparation [38]. Consequently, the dogma of the necessity of controlling intraoperative blood pressure fluctuations by PMP may not be challenged by objective data, whereas its initial argument was based on historical and methodologically biased studies. However, specialized actors taking direct charge of patients (anesthesiologists and surgeons) currently have more effective intraoperative therapeutic means than a possible PMP to operate safely on patients with pheochromocytoma.

### 3.6. Intraoperative Anesthetic Management

Intraoperative anesthetic management has become an important part of the care of patients with pheochromocytoma. This management requires a multidisciplinary team that has experience in adrenal cases. Communication between surgeons and anesthesiologists is crucial for good execution of surgeries. There are three critical intraoperative moments during an adrenalectomy for pheochromocytoma: orotracheal intubation, tumor dissection and phases after ligation of the main adrenal vein [53]. Hemodynamic intraoperative monitoring should include, in addition to basic monitoring such as an electrocardiogram, noninvasive BP, pulse oximeter and capnography in an artery so that there are real-time accurate measures of BP [53]. There is no precise protocol for anesthesia in patients with pheochromocytoma. The most important aspect to achieve is a deep anesthetic level so the cardiovascular system can be inhibited in response to the release of catecholamines [3]. There are several recommendations that should be followed: locoregional anesthesia should be avoided because it can cause the blocked regions to be more responsive to catecholamines, histamines should be avoided as they can release catecholamines from chromaffin granules, fentanyl should be preferred to morphine, propofol should be used instead of ketamine and anxiolytic drugs should be administered to patients before surgery to minimize stress stimuli [53].

From the point of view of the anesthesiologist, several different complications can occur during an adrenalectomy. Hypertension crises are usually related to manipulation of the tumor [53]. SPB > 200 mmHg for more than 1 min requires treatment [53]. It is probably not necessary to have a reaction from the anesthesia team below this limit. Several intraoperative drugs can be used, such as nitroglycerin (nitric oxide modulators), nicardipine (calcium channel blockers), phentolamine (alpha-adrenergic antagonist) or even magnesium sulphate to lower blood pressure [53]. Arrythmias may also happen due to the release of epinephrine or norepinephrine during manipulation of the tumor. It can manifest itself by either bradycardia with hypertension or by tachyarrhythmia. After tumor removal, patients may experience episodes of severe hypotension due to normalization of catecholamine levels and contralateral downregulation. Anesthesiologists should take care to maintain a satisfying blood volume during the whole surgery to prevent hypotension episodes as much as possible after the excision of the tumor. If treatment is necessary, a bolus infusion of crystalloids can be rapidly initiated. If the hypotension is refractory, vasopressin can be used [53]. The action of vasopressin has the advantage that it is not dependent on adrenergic receptors [53,54]. In current clinical practice, more than 95% of patients who undergo an adrenalectomy for pheochromocytoma can be discharged the day after surgery without going through an intensive care unit.

### 3.7. Clinical Guidelines: The Need for Evolution

The Clinical Endocrine Guidelines of 2014 recommend that patients with pheochromocytoma undergo a preoperative blockade to prevent perioperative cardiovascular complications [19]. This recommendation was validated by the 2020 Working Group on Endocrine Hypertension of the European Society of Hypertension [10]. Boutros et al. were the first to question the role of PMP and showed in the 1990s that an adrenalectomy for pheochromocytoma could be performed without preparation [55]. Since then, several studies have put focus on assessing the efficacity of PMP [47]. Given the many limitations of the studies available to date and the long experience with preoperative administration of antihypertensive drugs (mainly alpha blockers), the use of these drugs before surgery is still officially recommended. However, several centers have already stopped preparing patients before an adrenalectomy for pheochromocytoma. The two questions to consider now are: is it risky to continue preparing patients? And is it dangerous to stop preparing them? At first, some studies show that preparation with alpha blockers can favor certain complications (hypotension, orthostatic dysregulation, sustained arrhythmia), but this is not consensual [3,38,39]. To the second question, the answer is probably no. However, this answer cannot be provided by a randomized study [39]. The centers that have stopped preparing patients will be able to provide an answer in the months and years to come, but they will always be criticized for not having enough power to avoid an underestimation bias (because of the low incidence of complications). Registry data may be a solution, but again, reporting bias will be an issue, even if there are audits to evaluate them. We believe that the most important factor that the caregivers who actually manage patients with pheochromocytoma in the operating room (anesthesiologists, surgeons) should be able to evaluate their results and then share them with the aim of improving practices and the quality of patient management.

## 4. Conclusions

To conclude, the management of pheochromocytoma has significantly changed over the last decades, leading to a drastic decrease in morbi-mortality. Consequently, the use of PMP to improve intraoperative hemodynamic instability should be put into question because preoperative blood pressure normalization does not improve postoperative outcomes in current clinical practice. The current recommendations should be upgraded, and the role of anesthesiologists during an adrenalectomy for pheochromocytoma should be defined better and evaluated.

## Figures and Tables

**Table 1:** Comparison of laparoscopic versus open adrenalectomies for pheochromocytomas.

Author	Year	Design	LA/OA	LA Type	OT (min)		Blood Loss (mL)		Hospital Stay (Days)		CNS (*n*)		CONV (*n*)
					LA	OA	LA	OA	LA	OA	LA	OA	LA to OA
Vargas [20]	1996	RTP	6/6	Trans	193	178	245	283	3.1 ***	7.2 ***	NA	NA	1
Sprung [33]	1999	RTP	14/20	Trans	177	196	100 ***	400 ***	3 ***	7.5 ***	NA	NA	1
Schell [21]	1997	RTP	4/7	Trans	NA	NA	NA	NA	1.7 ***	7.8 ***	0	3	NA
Ichikawa [34]	2001	RTP	7/11	Trans Retro	145	165	55 **	330 **	12	14	1	3	1
Tanaka [24]	1998	RTP	10/7	Trans	240	288	200	400	8 **	15 **	1	2	1
Inabnet [28]	1997	RTP	11/11	Trans	146	153	NA	NA	5.5	6.1	0	1	0
Möbius [29]	1997	RTP	9/9	Trans	243 **	100 **	NA	NA	6 **	10 **	1	4	2
Kim [36]	2002	RTP	15/9	Trans	171	200	189.5 *	397.1 *	5.6 ***	12.4 ***	0	4	NA
Toniato [25]	2005	RTP	40/24	NA	78 **	149 **	100 *	200 *	3.7 **	10.1 **	1	4	NA
Kazaryan [31]	2002	RTP	9/22	Trans	132 *	129 *	178 *	420 *	3.2 *	9.2 *	0 *	3 *	NA
Tibierio [27]	2006	RCT	13/9	Trans	158	180	48 *	164 *	5 *	8 *	0	0	NA
Kasahara [30]	2007	RTP	23/18	Trans	210	212	120 *	400 *	9 **	19 **	0 **	4 **	4
Wang [23]	2013	RTP	23/28	Trans	158 **	121 **	47	102	4.2 **	9.7 **	2	2	2
Bai [26]	2017	RTP	82/100	Trans	167	150	100**	400**	9.8	10	19	36	NA
Fang [35]	2017	RTP	89/26	Trans	157 *	260 *	134 *	439 *	3.7 *	5.2 *	6	5	NA
Agarwal [32]	2010	RTP	49/52	Trans	270	258	223 **	473 **	6.1	10.4	3 **	12 **	19

* *p* < 0.05; ** *p* < 0.01; *** *p* < 0.0001; LA: laparoscopic adrenalectomy; OA: open approach; RTP: retrospective RCT: randomized controlled trial; Trans: transperitoneal; Retro: retroperitoneal; CNS: complications; CONV: conversion; OT: operative time; NA: not available.

**Table 2:** Definitions of intraoperative hemodynamic instability (IHI) in the literature.

Study	Year	Definitions of Intraoperative Hemodynamic Instability (IHI)
Inabnet [28]	2000	Highest MAP Highest BP
Bruynzeel [42]	2010	SBP > 160 mmHg MAP < 60 mmHg
Shao [46]	2011	Highest BP Minutes SBP > 30% preinduction baseline Minutes SBP > 200 mmHg Lowest BP Minutes SBP < 30% preinduction baseline HR > 110 bpm HR< 50 bpm
Brunaud [43]	2014	SBP > 160 mmHg MAP < 60 mmHg
Kiernan [40]	2014	SBP > 200 mmHg HR > 110 bpm
Gaujoux [46]	2015	SBP > 150 mmHg SBP < 90 mmHg HR > 110 bpm
Livingstone [46]	2015	10 hypo/hypertensive episodes where the anesthesiologist had to respond with a vasoactive substance
Namekawa [48]	2016	SBP > 160 mmHg SBP < 90 mmHg Dose of catecholamine to maintain SBP > 90 mmHg
Brunaud [37]	2016	SBP > 160 mmHg SBP > 200 mmHg MAP < 60 mmHg SBP > 200 mmHg + MAP < 60 mmHg
Kwon [46]	2016	SBP > 160 mmHg HR > 100 bpm
Vorselaars [46]	2017	SBP > 160 mmHg SBP > 200 mmHg MAP < 60 mmHg SBP > 160 mmHg + MAP < 60 mmHg SBP > 200 mmHg + MAP < 60 mmHg Intravenous vasopressor + vasodilator.
Groeben [48]	2017	Highest BP SBP > 250 mmHg
Askasakal [46]	2018	SBP > 200 mhg SPB < 90 mmHg Use of vasoactive drugs
Buitenwerf [39]	2019	SBP > 160 mmHg MAP < 60 mmHg
Tian [46]	2019	SBP > 200 mmHg SPB < 80 mmHg HR > 120 bpm
Thompson [46]	2019	SBP > 200 mmHg SBP < 90 mmHg HR > 120 bpm HR < 50 bpm Use of vasopressors
Buisset [46]	2021	SBP > 160 mmHg SBP > 200 mmHg MAP < 60 mmHg SBP > 200 mmHg + MAP < 60 mmHg

IHI: intraoperative hemodynamic instability; SBP: systolic blood pressure; HR: heart rate; MAP: mean arterial pressure; BP: blood pressure.

**Table 3:** Comparison of intraoperative hemodynamics between patients with and without PMP.

Study	Year	Patients	Blockade		IHD		Intraoperative Hypotension		Mortality		Morbidity	
			Yes	No	Bl.	No Bl.	Bl.	No Bl.	Bl.	No Bl.	Bl.	No Bl.
Groeben [38]	2017	1860	1517 1108 (alpha) 348 (other)	343	Systolic BP > 250 mmHg		NA	NA	8	1	90	3
					64	25						
Goldstein [40]	1998	104	67	16	3 (4.5%)	0	2 (3%)	0	0	0	NA	NA
Groeben [52]	2016	303	110 98 (alpha) 23 (dox)	1	Systolic BP > 250 mmHg		51% *	38% *	NA	NA	NA	NA
					11	16						
Brunaud [43]	2012	155	151 41 (alpha) 110 (CCB)	4	Greatest SBP (mmHg)		Lowest SBP (mmHg)		0	0	Alpha 24% CCB 15%	NA
					Alpha 169 ** CCB. 198 **	163 **	Alpha 82CCB 82	94				
Shao [46]	2011	50	38 (dox)	21	Greatest SBP (mmHg)		Lowest SBP (mmHg)		NA	NA	NA	NA
					154	153	90	92				
Ulchaker [46]	1994	113	79 (alpha,CCB,dox,BB)	34	Greatest SBP (mmHg)		Lowest SBP (mmHg)		0	0	6	0
					192	198	NA	NA				

* *p* < 0.05; ** *p* < 0.001; Alpha: alpha blockage; CCB: calcium channel blockers; Dox: doxazosin; Bb: beta blockers; SBP: systolic blood pressure; IHD: intraoperative hemodynamic; Bl.: blockade; No Bl.: No blockade.
